# The Impact of War-Related Stress on Coronary Artery Disease Severity in War Survivors: A SYNTAX Study

**DOI:** 10.3390/ijerph18063233

**Published:** 2021-03-21

**Authors:** Hanna Al-Makhamreh, Dana Alkhulaifat, Abdallah Al-Ani, Baraa Mafrachi, Aseel Saadeh, Hashim Al-Ani, Amjad Bani Hani, Saif Aldeen AlRyalat

**Affiliations:** 1 Department of Internal Medicine, Jordan University Hospital, Amman 11942, Jordan; 2 School of Medicine, University of Jordan, Amman 11942, Jordan; danakhulaifat@gmail.com (D.A.); abdallahalany@gmail.com (A.A.-A.); baraawail101@gmail.com (B.M.); aseel.saadeh96@gmail.com (A.S.); hashimamer995@gmail.com (H.A.-A.); 3 Department of Surgery, Jordan University Hospital, Amman 11942, Jordan; amjadbh@yahoo.com; 4 Department of Ophthalmology, Jordan University Hospital, Amman 11942, Jordan; saifryalat@yahoo.com

**Keywords:** armed conflicts, coronary artery disease, refugees, stress, SYNTAX

## Abstract

Background: Due to the strong relationship between stress and heart disease, particularly acute myocardial infarction (MI), this study investigated the complexity of coronary artery disease (CAD) among Syrian refugee patients referred to Jordan University Hospital and its relation to war-related stressors. Methods: This is a retrospective study that utilized the SYNTAX I score in order to evaluate all Syrian refugees that underwent coronary artery catheterization at Jordan University Hospital during the period between May of 2014 and December of 2017. Results: There was a significant association between war-related stressors and high SYNTAX score (SX score), thus indicating a higher complexity of CAD in Syrian war survivors with higher stress scores. The strongest war-related correlation was observed with crossing green-lines, in which Syrian refugee patients who had crossed such lines had significantly higher SYNTAX scores. Regression analysis demonstrated that war stressors were positive predictors of increased SYNTAX scores even when adjusted for conventional CAD risk factors. Surprisingly, none of the CAD risk factors were significantly associated with SYNTAX score. Conclusion: Our findings suggest that exposure to multiple war-related stressors may increase the complexity and severity of CAD in Syrian war survivors. Thus, special attention, efforts, and resources should be allocated to screen for such vulnerable patients in order to provide them with the appropriate healthcare.

## 1. Introduction

Coronary artery disease (CAD) is widely regarded as the leading cause of morbidity and mortality in developed countries, accounting for at least one third of all deaths among individuals in the United States [1,2]. Similarly, for developing countries (which include the Middle East, India, China, Latin America, and Sub-Saharan Africa), morality was anticipated to double from 1990 to 2020, from 9 million to 19 million deaths [3]. While there are many risk factors for developing CAD, such as hypertension and smoking, some of the main contributors to the development and exacerbation of CAD are psychosocial factors [4]. Such factors include acute and chronic stress, which have been linked to CAD through multiple proposed pathophysiological processes [5]. Furthermore, chronic stress often manifests as post-traumatic stress disorder (PTSD), which has also been found to be an independent risk factor for the development of CAD [6,7].

Since 2011, the Syrian crisis subjected its natives to the horrid conditions of war, ultimately forcing large numbers of Syrians to flee to nearby countries, one of which is Jordan. It is estimated that more than 1.26 million Syrian refugees reside within Jordan, all receiving healthcare covered by the United Nations High Commissioner for Refugees (UNHCR) or its associate organizations [8]. Those affected by the atrocities of war experience a variety of stressors such as family separation, loss of loved ones, loss of homes, shelling, and a constant threat to life. Such traumatic experiences can go on to have tremendous psychological burden.

Prior to the 2011 civil war, almost half of all Syrian deaths were caused by cardiovascular diseases (CVD) [9]. Moreover, the Syrian refugee crisis has seen a climb in deaths due to non-communicable diseases (NCD), the most common of which are CVD and their respective risk factors. The mortality rates due to NCD are as similar to those inflicted by war. The mortality rates of both NCD and guerilla warfare have proved to be analogous [10]. A Jordanian study conducted on Syrian refugees found that 69.6% of refugees were found to have some form of CAD [8].

It has been proposed that when all traditional CAD risk factors were adjusted for, a significant association exists between war-related events and CAD [11]. The Syrian refugee population has experienced a variety of war-related stressors as well as difficulties in accessing health care [12]. Therefore, such population may be vulnerable to the long-term complications of CAD as precipitated by chronic and severe war stressors. This may influence policy makers to allocate resources for the screening and clinical evaluation of Syrian war survivors as to alleviate their healthcare costs in the long-term. The aim of this study is to evaluate whether war-related stressors contribute to the increased complexity of CAD among Syrian refugees using the SYNTAX I score [13,14].

## 2. Materials and Methods

### 2.1. Study Population and Sampling Technique

The study retrospectively examined the records of 340 Syrian refugee patients with various forms of CAD who underwent coronary catheterization with or without percutaneous coronary intervention (PCI) at Jordan University Hospital (JUH) from May 2014 to December 2017. Overall, a total of 249 patients were excluded from the study. Exclusion criteria included the following: patients younger than 18 years old, patients with inconclusive medical records, patients with missing angiographic records, patients with a previous history of coronary artery bypass graft surgery (CABG), patients that have not experienced any war-related event or are presently deceased. The study sampled its participants out of all Syrian refugee patients who had viable records of undergoing a coronary catheterization at JUH.

### 2.2. SYNTAX Score and Angiographic Analysis

SYNTAX scores (SX score) were calculated for all coronary lesions causing a ≥50% diameter stenosis in vessels ≥1.5 mm. Such calculation was made possible by utilizing the SYNTAX-1 algorithm found at (http://www.syntaxscore.com (accessed on 16 January 2020)). The SX score calculation are performed as follows: (1) selecting the dominant coronary tree of the patient, (2) identifying the lesions within the tributaries of the coronary tree’s major arteries including the right circumflex artery (RCA), left main artery (LM), left anterior descending (LAD), and left circumflex artery (LCX), (3) identifying the presence of total occlusions, trifurcations, bifurcations, severe tortuosity, heavy calcification and thrombi. Upon identification of the aforementioned, the algorithm stated above calculates the patient’s SX score.

Two independent cardiologists analyzed the angiograms and calculated the SX scores for more than 140 cases. The cardiologists were double blinded to both patients’ characteristics and the outcomes of angiogram readings of their counterpart. When in disagreement, a third observer is consulted and the final result is reached by consensus. Such protocol accounts and limits intra- and inter-observer bias. Cases were stratified into three major SX score categories: Low SX score (≤22); Intermediate SX score (23–32); and High SX score (≥33). The validity and reliability of the SX score as a predictor of cardiac complications and CAD complexity was previously demonstrated in stage 3 clinical trials and meta-analyses [13,14].

### 2.3. Data Collection

A team of researchers extracted and evaluated the refined cases based on the aforementioned inclusion and exclusion criteria. The evaluation process targeted the patient’s baseline characteristics in an effort to match the studied cases and limit the effect of preliminary confounding variables. All patients were approached through telephone interviews that followed a scripted questionnaire with exclusively closed ended questions. A unique questionnaire was used in this cohort study to evaluate patients’ demographics, medical history, and war-related stressors. The war-related stressors evaluated were restricted to neighborhood dissatisfaction, living in dangerous locations, and crossing green-lines (demarcation lines separating hostile forces). The cohort of patients was categorized based on the amount of war-related stressors they faced into 4 categories: Severe war stress (≥3 stressors); moderate war stress (2 stressors); mild war stress (1 stressor); and no war stress (zero stressors). The items in the questionnaire were influenced by the available literature regarding war stressors and CAD [11]. Of the contacted 91 eligible participants, 100% responded to our calls and agreed to participate in the study and answered the questionnaire’s items.

### 2.4. Statistical Analysis

The study results are reported in the form of descriptive statistics. Means and standard deviations (SD) described continuous variables (e.g., age). Frequencies were used to describe other nominal variables (e.g., gender). Chi-Square and Fischer tests were utilized to investigate the association between stress score categories with SX scores, and other categorical variables. Binning was used to categorize continuous SX scores. Linear regression analysis was conducted to demonstrate predictors of higher SX scores. The model’s R^2^ is 0.81, while its Durbin–Watson statistic value is 2.030. Any association that has a P-value of 0.05 or less is considered statistically significant. All associations are reported with 95% confidence level and an alpha value of 5%. All tests and processes are conducted on the Statistical Package for Social Sciences (SPSS) version 21 (Chicago, IL, USA).

### 2.5. Ethical Considerations

The study’s protocol was approved by both JUH’s ethics committee and University of Jordan’s Institutional Review Board (IRB) (6486/2019/67). All contacted patients agreed verbally on a scripted consent form that explains the premise of the study, their rights, and the taken measures that enforce absolute confidentiality of their data. The study’s protocol is in line with the ethical guidelines delineated in the 1975 Declaration of Helsinki.

## 3. Results

The study recruited a total of 91 Syrian refugee patients who underwent coronary catheterization with or without PCI at JUH between May 2014 and December 2017. The mean age of the study population is 56 (SD: 10.01) years, with a clear male dominance (M: F, 73.6%: 26.4%). The average BMI of our sample is 29.4 (SD: 6.29). The majority of patients reside in urban areas (80.2%) while the rest reside in refugee camps (19.8%).

All 91 patients were referred to JUH for acute coronary syndrome as follows, 69 (75.8%) were diagnosed with unstable angina, 18 (19.8%) STEMI, and 4 (4.4%) NSTEMI. In term of concomitant co morbidities and risk factors, 81 (89.0%), 48 (52.7%), and 74 (81.3%) have an established diagnosis of hypertension, diabetes mellitus, and hyperlipidemia, respectively. More than half of the participants are smokers (58.2%) and almost 48.4% had peripheral arterial disease. Moreover, 55 (60.4%) patients had an identifiable family history of CAD, 26 (28.6%) had previous history of myocardial ischemia, and 25 (27.5%) had prior PCI. In terms of coronary catheterization reports, 37 (40.7%) patients had single vessel disease, 31 (34.1%) had two-vessel disease, and 18 (19.8%) had three-vessel disease. Patients’ clinical characteristics are reported in Table 1.

SX score was calculated for the all 91 patients. SX score ranged from 1 to 54.5, with a mean of 17.41 (SD: 12.50). Among the study’s sample, 65 (71.4%) patients were stratified in Low (SX score ≤ 22) group, 13 (14.3%) patients in Intermediate (23 > SX score ≤ 32) group, and 13 (14.3%) patients in High (SX score ≥ 33) group (Table 2).

Our survey of the studied population illustrated that, the most experienced war stressors by Syrian refugees were the following: residing in active war zones (dangerous locations) (83.5%), crossing green lines infested with mines and sniper fire (46.1%), and feeling dissatisfied with their neighborhoods (71.4%). Patients were stratified into four groups based on the cumulative number of experienced war-related stressors: no war stress group (*n* = 6, (6.6%)); low war stress group (*n* = 14, (15.4%)); moderate war stress group (*n* = 44 (48.3%)); and high war stress group (*n* = 27 (29.7%)). The four groups did not differ in term of comorbidities (*p* = 0.103), ischemic events (*p* = 0.993), BMI (*p* = 0.079).

**Figure 1:**
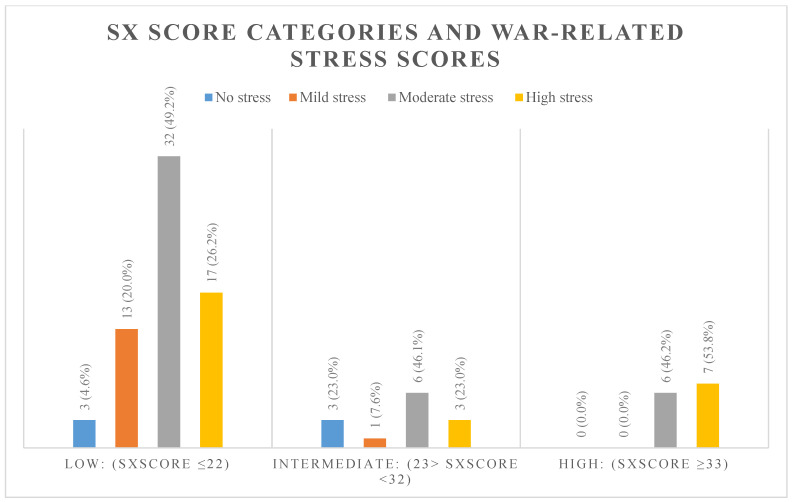
Frequency of war-related stress degrees within the different SX score categories.

**Figure 2:**
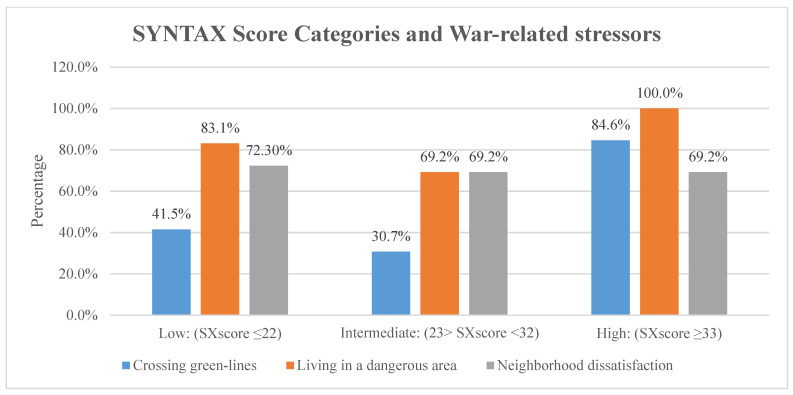
Percentage of war-related stressors within each SX score category. Chi-square testing shows a significant association between only crossing green-lines and SX score categories. Crossing green-lines (*p* < 0.01), living in a dangerous area (*p* = 0.11), and neighborhood dissatisfaction (*p* = 0.96).

Upon comparing the SX score with the frequency of experiencing war-related stressors, we found a significant association between SX score and war-related stress score (*p* = 0.04). Approximately 53.8% of those with high war stress had a high SX score, compared to 46.2% with a moderate war stress score (Refer to Figure 1). The most significant association was seen between SX score and crossing green lines (*p* < 0.01), where 84.6% of those who had High SX score crossed green lines compared to 41.5% of those with Low SX score (Refer to Figure 2). Finally, no statistically significant difference was found between the three SYNTAX groups with regard to hypertension (*p* = 0.37), diabetes mellitus (*p* = 0.29), hyperlipidemia (*p* = 0.87), smoking (*p* = 0.29), PAD (*p* = 0.49), and family history of CAD (*p* = 0.27). Moreover, linear regression analysis showed that stress is a positive predictor of higher SX score categories (ß: 4.411; *p*-value: >0.01; 95% CI: 1.49–7.32). All other risk factors and comorbidities were insignificant predictors of SX score on the regression model. Table 3 demonstrates the regression model utilized in the study.

## 4. Discussion

Our results demonstrated that higher war stress variables were significantly associated with a higher SX score (*p* = 0.04). Such observations indicate that patients exposed or subjected to higher war stressors exhibit higher rates of mortality and major adverse cardiac events (MACE) which include myocardial infarction, stroke, vessel revascularization, and all-cause death [15]. The SX score is an objective anatomic tool for the assessment of coronary complexity [16]. It is an effective tool for the prediction of risk of MACE and mortality trends, particularly at its highest score tertile. Moreover, its predictive capabilities are validated in patients who underwent PCI [17,18]. Similar results were reported in the literature, in which a study conducted on cardiac patients during the Lebanese civil war illustrated that the presence of a significant association between war stressors and CAD [11]. However, our study utilized a reliable scoring system to accurately assess the impact of stress on the complexity of CAD.

The influence of stress on the development and worsening of cardiac disease has been well established in the literature [4,19,20]. Stress is associated with CAD through different intertwined mechanisms. Stressful conditions subject the body to a ‘fight or flight’ state, characterized by sympathetic overstimulation, pro-inflammation, and endothelial dysfunction [4,21,22,23]. Increased levels of catecholamines mediates hypertension, increased myocardial demand and decreased myocardial perfusion, all of which are associated with platelet aggregation, plaque rupture, rhythmic disorders, and perpetual coronary vasoconstriction [4,24]. In addition, a pro-inflammatory state is established through increased inflammatory cells, elevated endothelial adhesion molecules, and an influx of chemotactic agents. Such state will consequently promote early atherosclerotic plaque development and endothelial dysfunction of coronaries [21]. As a result, stress induces myocardial ischemia, arrhythmogenesis, and hypercoagulation within the coronary vasculature [23]. Furthermore, studies show that stressful conditions could improperly activate and impair the brain’s cardiac regulatory cortical areas and even induces cardiac remodeling and electrophysiological alterations which might accentuate cardiac disease [25,26].

Out of the evaluated war stressors, crossing green-lines showed significant association with higher SX score (*p* < 0.01). Crossing green lines is a life-threatening experience that precipitates severe psychological trauma due to the menacing nature of such lines. Those areas harbor snipers, cannons, and multiple checkpoints, all of which belong to different political and military parties, that fire and operate without any form of warning. This result is consistent with studies conducted during war times in Lebanon and Croatia, in which the mere transportation of patients during war time contributed to higher stress, thereby contributing to development of heart disease [11,27].

Affective disorders characterized by immense psychological trauma, such as war-related PTSD, are associated with cardiovascular disorders [6,28]. Such disorders exert their effect through sympathetic overstimulation, hypothalamic-pituitary-adrenal axis dysregulation, and promoting immune-inflammatory dysfunction [6,7,28]. Furthermore, PTSD is associated with behavioral alternations such as increased smoking or increased alcohol consumption, which acts as potentiating factors to CAD development [29,30]. In addition to psychological stress, external cues to stress such as low socioeconomic status and the lack of reliable refugee support programs might have contributed to higher stress levels or advocated unhealthy behaviors associated with cardiovascular events [23].

This study did not show any significant association between CAD risk factors that were studied (hypertension, diabetes, dyslipidemia, family history of cardiac diseases, peripheral arterial diseases, and prior myocardial infarction) with the different categories of SX score. Likewise, there were no statistical differences between different stress groups and the comorbidities mentioned above. This observation further elucidates the effects of war stressors on the increasing complexity of CAD in Syrian refugees as indicated by the high SX score in the high stress group. The aforementioned observation was consistent with the regression analysis, in which only stress was a positive predictor of increasing SX score when all CAD risk factors were accounted for.

In other words, chronic stressors such as those related to war significantly contribute to higher rates of mortality of Syrian refugee patients visiting JUH. This population whom experienced war-related stressors are at a significant risk of developing long term complications such as CAD and increased rates of major adverse cardiac events (MACE) irrespective whether they were subjected to percutaneous coronary intervention (PCI) or not. Besides, such vulnerable population could be exposed to an array of acute coronary events [24]. This calls for clinical evaluation of the psychological profiles of Syrian war survivors and tending to their social needs, in order to anticipate or predict the severity of their newly developed or preexisting CAD [23,24]. In fact, mental stress-induced ischemia is predominantly silent and not even associated with electrophysiologic or crude angiographic changes [31]. Considering the aforementioned points, psychological interventions should be offered to these vulnerable populations as they have proven their effects in controlling rates of cardiac risk and prolonging life [32,33].

### Limitations

Our findings should be interpreted with caution due to a plethora of limitations. The study did not utilize a comparative control group when assessing the effects of stress on cardiac mortality. Referral bias to coronary angiography might underestimate our sample due to the inconsistent indications criteria used by cardiologists and the limited resources allocated to the refugee population which might have prevented them from undergoing the operation. Moreover, recall bias is suspected as reports of stress variables could be affected by the patients’ socioeconomic and health status. Finally, the study’s limited sample size might have greatly impacted its statistical power. Nonetheless, it should be considered that all of the study participants are refugees who live in remote areas, fail to sustain follow-up, and have limited expenses.

## 5. Conclusions

Our study utilized the SYNTAX I score to evaluate CAD complexity in vulnerable Syrian refugee patients who were subjected to severe war stressors. Our results demonstrated that chronic war-related stressors significantly contributed to higher complexity and severity of CAD in Syrian refugee patients. Such association emphasizes the vulnerability of the population living through severe stress, generated from the surrounding war-related experiences. Thus, further efforts should be made in order to screen for such high-risk patients. Moreover, resources should be allocated in order to evaluate those patients mentally and physically. Nonetheless, larger multi-central studies should be conducted to assess the impact of war stressors on the clinical pictures of refugees and war survivors. Future studies should strive to use objective surrogate markers of stress (e.g., hair cortisol, lipoprotein (a)) in order to elucidate the effects of acute and chronic stressors on the cardiac complexity of susceptible patients. Moreover, cardiac complexity might be better examined with the SYNTAX II score as it accounts for the clinical variables that may confound the relationship between stress and cardiac pathology.

## Figures and Tables

**Table 1:** Demographic profile and clinical characteristics of the study population.

Variables	Category	Low SX Score	Intermediate SX Score	High SX Score	*p* Value *
Gender	Male	10 (14.9%)	10 (14.9%)	47 (70.1%)	0.90
	Female	18 (75.0%)	3 (12.5%)	3 (12.5%)	
Age (Years)	18–44	11 (84.6%)	1 (7.7%)	1 (7.7%)	0.45
	45–64	41 (70.7%)	10 (17.2%)	7 (12.1%)	
	≥65	13 (65.0%)	2 (10.0%)	5 (25.0%)	
BMI	Normal	15 (75%)	2 (10.0%)	3 (15.0%)	0.94
	Overweight	25 (67.6%)	6 (16.2%)	6 (16.2%)	
	Obese	25 (73.5%)	5 (14.7%)	4 (11.8%)	
Location of residence	Urban	53 (72.6%)	10 (13.7%)	10 (13.7%)	0.88
	Refugee Camp	12 (66.7%)	3 (16.7%)	3 (16.7%)	
Income ^†^	Low < 300	57 (70.4%)	12 (14.8%)	12 (14.8%)	0.81
	Intermediate 300–600	8 (80.0%)	1 (10.0%)	1 (10.0%)	
Co-morbidities	Hypertension	57 (70.4%)	11 (13.6%)	13 (16.0%)	0.37
	Diabetes Mellitus	34 (70.8%)	5 (10.4%)	9 (18.8%)	0.28
	Dyslipidemia	52 (70.3%)	11 (14.9%)	11 (14.9%)	0.87
	Smoker	40 (75.5%)	5 (9.4%)	8 (15.1%)	0.29
	Peripheral Arterial Disease	31 (70.5%)	5 (11.4%)	8 (18.2%)	0.49
	Family History of CAD	30 (70.9%)	6 (10.9%)	10 (18.2%)	0.27
History of previous myocardial ischemia		17 (65.4%)	3 (11.5%)	6 (23.1%)	0.30
History of previous Percutaneous Coronary Intervention (PCI)		16 (71.4%)	13 (14.3%)	13 (14.3%)	0.26

* *p* value indicates associations between different variables and SYNTAX categories. ^†^ Income is reported in Jordanian Dinar (JD). 1 JD = 1.41 USD.

**Table 2:** SX score and war stress score crosstabulation.

War Stressors	Total n (%)	Low: (SX Score ≤ 22)	Intermediate: (23 > SX Score < 32)	High: (SX Score ≥ 33)	*p* Value
No Stress	6 (100%)	3 (50.0%)	3 (50.0%)	0 (0.0%)	0.042
Low Stress	14 (100%)	13 (92.9%)	1 (7.1%)	0 (0.0%)	
Moderate Stress	44 (100%)	32 (72.7%)	6 (13.6%)	6 (13.6%)	
High Stress	27 (100%)	17 (63.0%)	3 (11.1%)	7 (25.9%)	

**Table 3:** Regression model for predictors of SX score.

Variable	Beta Coefficient	*p*-Value	95% CI
Age	0.026	0.847	(−) 0.247–0.299
BMI	(−) 0.225	0.151	(−) 0.609–0.098
Smoking status	(−) 6.061	0.059	(−) 11.796–(−) 0.326
Diabetes Mellitus	0.943	0.690	(−) 3.811–5.698
Hypertension	(−) 3.307	0.511	(−) 13.414–6.801
Dyslipidemia	3.441	0.208	(−) 2.003–8.885
Family History of CAD	2.993	0.179	(−) 1.435–7.421
Prior MI	1.712	0.649	(−) 5.845–9.269
Prior PCI	(−) 0.806	0.827	(−) 8.247–6.635
PAD	(−) 1.938	0.467	(−) 7.279–3.403
War Stressors	4.411	0.004	1.496–7.326
1-vessel disease	(−) 2.770	0.559	(−) 12.304–6.764
2-vessel disease	2.193	0.664	(−) 7.958–12.345
3-vessel disease	1.229	0.816	(−) 9.383–11.841
Number of stents	(−) 1.069	0.494	(−) 4.208–2.070
Lesions ≥ 20 mm	(−) 2.294	0.377	(−) 7.495–2.908

The R^2^ for this model is 0.81. Durbin–Watson statistic value for this model is 2.030.

## Data Availability

All data contributing to the conclusions found in this paper are available upon request from the authors.
